# Piccolipiù, a multicenter birth cohort in Italy: protocol of the study

**DOI:** 10.1186/1471-2431-14-36

**Published:** 2014-02-07

**Authors:** Sara Farchi, Francesco Forastiere, Liza Vecchi Brumatti, Sabrina Alviti, Antonio Arnofi, Tommaso Bernardini, Maura Bin, Sonia Brescianini, Valentina Colelli, Rodolfo Cotichini, Martina Culasso, Paolo De Bartolo, Laura Felice, Valentina Fiano, Alessandra Fioritto, Alfio Frizzi, Luigi Gagliardi, Giulia Giorgi, Chiara Grasso, Francesca La Rosa, Claudia Loganes, Paola Lorusso, Valentina Martini, Franco Merletti, Emanuela Medda, Veronica Montelatici, Isabella Mugelli, Silvia Narduzzi, Lorenza Nisticò, Luana Penna, Elisa Piscianz, Carlo Piscicelli, Giulia Poggesi, Daniela Porta, Antonella Ranieli, Gherardo Rapisardi, Assunta Rasulo, Lorenzo Richiardi, Franca Rusconi, Laura Serino, Maria Antonietta Stazi, Virgilia Toccaceli, Tullia Todros, Veronica Tognin, Morena Trevisan, Erica Valencic, Patrizia Volpi, Valentina Ziroli, Luca Ronfani, Domenico Di Lallo

**Affiliations:** 1 Laziosanità- Agenzia di Sanità Pubblica della Regione Lazio (Public Health Agency, Lazio Region, Italy), Rome, Italy; 2 Dipartimento di Epidemiologia del SSR del Lazio-ASL RME, Rome, Italy; 3 Institute for Maternal and Child Health – IRCCS “Burlo Garofolo”, Via dell’Istria 65/1, Trieste 34137, Italy; 4 CNESPS - Istituto Superiore di Sanità, Rome, Italy; 5 Ospedale Cristo Re dell’Istituto Figlie di Nostra Signora a Monte Calvario, Roma, Italy; 6 Casa di Cura Città di Roma, Rome, Italy; 7 Department of Woman and Child Health, Ospedale Versilia, Local Health Authority 12, Viareggio, Italy; 8 Cancer Epidemiology Unit-CeRMS, Department of Medical Sciences, CPO-Piemonte, University of Turin, Turin, Italy; 9 Santa Maria Annunziata Hospital, Bagno a Ripoli, Italy; 10 Unit of Epidemiology, “Anna Meyer” Children’s University Hospital, Florence, Italy; 11 Maternal-Fetal Medicine Unit, Department of Surgical Sciences, AO Città della Salute e della Scienza di Torino, University of Turin, Turin, Italy; 12 Institute of Clinical Physiology, National Research Council, Pisa, Italy; 13 School of Specialization in Hygiene and Preventive Medicine, Tor Vergata University, Rome, Italy

**Keywords:** Birth cohort, Early-life exposure, Infant and child health and development

## Abstract

**Background:**

The fetal and infant life are periods of rapid development, characterized by high susceptibility to exposures. Birth cohorts provide unique opportunities to study early-life exposures in association with child development and health, as well as, with longer follow-up, the early life origin of adult diseases. Piccolipiù is an Italian birth cohort recently set up to investigate the effects of environmental exposures, parental conditions and social factors acting during pre-natal and early post-natal life on infant and child health and development. We describe here its main characteristics.

**Methods/design:**

Piccolipiù is a prospective cohort of expected 3000 newborns, who will be recruiting in six maternity units of five Italian cities (Florence, Rome, Trieste, Turin and Viareggio) since October 2011. Mothers are contacted during pregnancy or at delivery and are offered to participate in the study. Upon acceptance, their newborns are recruited at birth and followed up until at least 18 years of age. At recruitment, the mothers donate a blood sample and complete a baseline questionnaire. Umbilical cord blood, pieces of umbilical cord and heel blood spots are also collected. Postnatal follow-up currently occurs at 6, 12, and 24 months of age using on-line or postal self administered questionnaire; further questionnaires and medical examinations are envisaged. Questionnaires collect information on several factors, including mother’s and/or child’s environmental exposures, anthropometric measures, reproductive factors, diet, supplements, medical history, cognitive development, mental health and socioeconomic factors. Health promotion materials are also offered to parents.

**Discussion:**

Piccolipiù will broaden our understanding of the contribution of early-life factors to infant and child health and development. Several hypotheses on the developmental origins of health can be tested or piloted using the data collected from the Piccolipiù cohort. By pooling these data with those collected by other existing birth cohorts it will be possible to validate previous findings and to study rare exposures and outcomes.

## Background

Exposures that act in early life, from the periconceptional period to the first years of life, affect normal growth, development and health in childhood and across the life course [1,2].

Starting from Barker’s hypothesis and the concept of developmental origins of health and disease [3], there is accumulating evidence of a relationship between early life exposures and a wide range of chronic diseases in children and adults, including cardiovascular and respiratory diseases, impaired neurodevelopment, and cancer.

Early-life factors that might potentially affect future health can act during or before the period of fetal development (endocrine disruptors, maternal diet, maternal smoking or alcohol consumption, occupation, social position), during infancy (breast- or formula feeding, weaning, exposure to indoor and outdoor pollutants, growth patterns, and development) and during childhood or adolescence (diet and levels of physical activity, environmental tobacco smoke (ETS), active smoking and alcohol consumption, psychological status) [1].

Fetuses, infants and young children are particularly vulnerable to the effects of early life exposures due the critical windows of vulnerability that occur during the rapid growth and development of organs and systems, to the immaturity of their metabolism, and, in the case of environmental exposures, to the greater intake and absorption of toxic hazards in children in relation to their body weight [4].

Birth cohort studies are a powerful study design for medical and social research, because they are designed to observe the impact of early exposures prospectively and at multiple time points during child development. Birth cohort studies allow for the collection of biological material from mothers and children, for the measurement of biomarkers and for the study of genetic and epigenetic factors.

In Italy, a number of projects are already collecting information during pregnancy or on newborns at birth and following them up through childhood and adolescence. Among existing birth cohort studies there are: two multipurpose hospital-based cohort studies -GASPII in Rome and CONER in Bologna [5], a national internet-based multipurpose cohort (NINFEA) [6], the Multiple Births Cohort Study (MUBICOS) [7] run within the Italian Twin Registry (ITR) [8], and the Trieste child development cohort [9].

Researchers working on the above listed projects are also collaborating to the Piccolipiù birth cohort project. The project brings together Italian expertise on birth cohort research and, in addition to its research objectives, is aimed at promoting healthy lifestyle, using innovative tools. Piccolipiù is a multipurpose birth cohort set up in multiple sites, in central and northern Italy. A biobank has also been developed, as part of the Piccolipiù project, in order to collect and store maternal and child biological samples.

The Piccolipiù birth cohort was designed in 2010, in a period of acute economic crisis, especially in Italy. Attention was therefore paid to the role of socioeconomic factors to understand how rapid economic and work-related changes can affect health in the long-term, and to identify possibilities for interventions that may mitigate the risks [10].

The main research objectives of this multipurpose birth cohort study are:

– to investigate the association between genetic, obstetric, socioeconomic, environmental and lifestyle characteristics and risk factors and infant and childhood morbidity and development;

– to describe the complex interactions between genetic, epigenetic, lifestyle factors and the environment;

– to promote infant and child health using traditional and innovative tools (e.g. social media) to communicate evidence-based prevention messages.

## Methods/design

**Figure 1:**
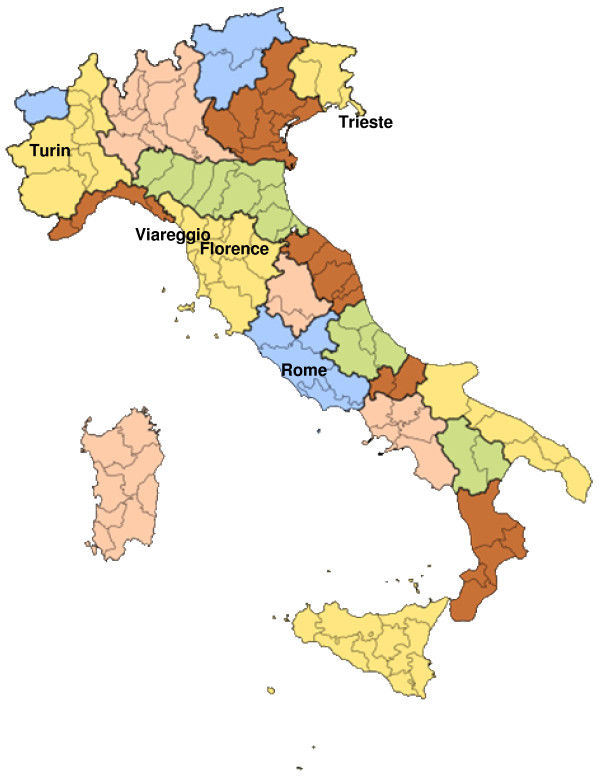
Geographical distribution of Piccolipiù cohort in Italy.

The Piccolipiù study plans to recruit and follow-up a birth cohort of 3000 children born in selected maternal units located in five Italian cities (Florence, Rome, Trieste, Turin, and Viareggio). Figure 1 shows the geographical distribution of the recruiting centers. The coordination of the Piccolipiù study is provided by a steering committee consisting of representatives of each study centre, of the centre in charge of data management and of the centre in charge of the Piccolipiù biological bank. The study was approved and initially funded by the Italian National Centre for Disease Prevention and Control (CCM grant 2010) and by the Italian Ministry of Health (art 12 and 12bis D.lgs 502/92).

### Inclusion and exclusion criteria

All singleton pregnant women giving birth in one of the selected maternity units are eligible for recruitment if they:

1. Are at least eighteen years old;

2. Are resident in the catchment area of the maternity centers;

3. Have enough knowledge of the Italian language to adequately understand the informed consent and to complete the questionnaires;

4. Have at least a telephone number they can be reached at.

### Recruitment

Recruitment started in October 2011 and is planned to continue throughout 2014. All pregnant women and their partners are invited to participate in the study at different times during the pregnancy: in Florence, Trieste, Rome, Viareggio they are contacted during antenatal classes, routine visits to prenatal clinics or ultrasound examinations at 26–34 weeks of gestation; in Turin at admittance to the hospital for delivery. Women receive an information leaflet, explaining the purposes of the project and the family commitments. Trained project personnel (i.e., midwives, physicians, psychologists, research nurses or research assistants) explain the study, enroll eligible women and supervise the completion of the baseline questionnaire.

A unique study identification number is assigned to each woman-child pair. This number identifies and tracks the questionnaires and the biological samples collected during the study to make them reversibly anonymized. This procedure is fundamental for linking information, ensuring confidentiality, and personal data protection according to Italian law.

### Follow-up

Mothers are contacted at 6, 12 and 24 months after delivery to collect follow-up information through questionnaires. One or two weeks before the scheduled date mothers receive a letter in which they are invited to fill in the questionnaire. Non respondents are repeatedly solicited by e-mail, SMS, telephone or regular mail following a predefined protocol. Further follow up is planned every 2 to 3 years by means of questionnaires and medical examination in an outpatient clinic.

### Questionnaires

The main topics explored by the study questionnaires are described in Additional file 1.

The baseline questionnaire is self-administered, except for the sections regarding maternal health during pregnancy, which are completed by a face to face interview with trained personnel after birth, and for the information on the delivery which is obtained directly from the hospital charts. Study personnel also check whether self-administered sections have been fully completed and if they need to be clarified so that the mother can give more precise and exhaustive answers. The follow-up questionnaires are also self-administered. Mothers are given the opportunity to complete either an on-line or a paper version of the questionnaires. In few cases, the possibility of a telephone interview is also offered (e.g. to hard non-responders).

Data are stored in a centralized database held by the data management centre. In order to enhance the quality of the data, the online questionnaires include consistency and range checks to prevent internal inconsistencies (e.g.: simultaneous presence of exclusive breastfeeding and introduction of solid foods). Furthermore, the on-line questionnaires are divided in several sections and a warning against missing values is given to the respondent every time a new section is submitted.

For paper questionnaires, quality of data is checked by the study personnel and, in case of inconsistent answers, mothers are contacted by phone to complete or correct the answers.

### Biologic samples

Biological material is collected, processed and temporarily cryopreserved in the maternity centers and periodically transferred to the Piccolipiù biobank, located at the Istituto Superiore di Sanità (ISS, Italian National Institute of Health), for final storage. Blood samples (4 ml in EDTA and 5 ml in serum separator tubes) are drawn from mother’s cubital vein just before or after delivery, and from the child’s umbilical cord soon after birth. In addition, three small pieces of umbilical cord are stored in 1.5 ml tubes and frozen within 24 hours. Finally, during the compulsory neonatal screening test at 48 hours of life, additional heel blood is spotted on filter paper (WhatmanTM Protein saver cards cat. 10534320) and let dry at room temperature.

Blood tubes are stored at 4°C for less than 24 hours, until they are centrifuged at 4°C or at 20°C for 10 minutes at 1300 gravities. Blood in serum separator tubes is allowed to clot at least 30 minutes before centrifugation. Fractionated EDTA blood is dispensed in 4 aliquots of plasma (≈0.5 ml), 1 aliquot of at least 0.1 ml of white blood cells (buffy coat) and 1 ml aliquot of erythrocytes. Serum is divided into 4 aliquots of approximately 0.5 milliliters. Cryotubes with cord and blood derivatives are periodically shipped with dry ice to the Piccolipiù biobank where they are stored in a nitrogen vapor tank and in -80°C freezers with nitrogen backup system. Filter cards are also transferred to the central biobank for long-term storage at -80°C in sealed bags. Incomplete sample collection is not considered as exclusion criteria for follow up.

The Piccolipiù biobank has a system for recording the exact location of each sample and its storage conditions.

### Health promotion materials

**Figure 2:**
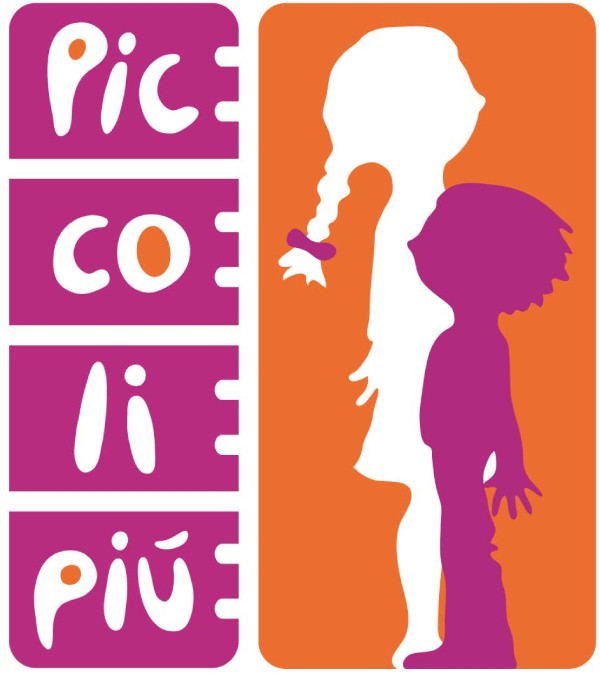
The Piccolipiù study logo.

During the study, different tools are used to convey health promotion messages to the participating families. All these tools are marked with the Piccolipiù logo, depicted in Figure 2. Before the newborn is discharged from the hospital, the mother receives a booklet containing evidence-based health promotion messages for infants in the first year of life. In particular, the booklet contains suggestions on breastfeeding, safe sleep position, avoidance of smoking, safe home environment, use of safety seats and other security measures for car transport, vaccinations. Furthermore, in order to help the completion of the follow up questionnaires, the booklet contains forms in which the parents can record on a monthly basis the child’s weight and length, information on breastfeeding, weaning, sleeping patterns, and possible diseases or symptoms.

At the time of completion of the 12^th^ month follow up questionnaire, mothers receive another booklet containing evidence-based health promotion messages for children from one to three years of age (e.g. use of safety seats and other security measures for car transport, reading aloud, sun protection, etc.). Again, mothers can use the booklet to record data on the child’s growth and on the milestones reached by the baby.

At the time of completion of the 24 months follow up questionnaire, mothers receive a book of recipes, edited by the Unit for Health Services Research of the Institute for Maternal and Child Health IRCCS Burlo Garofolo and developed within a project promoting healthy diet and physical activity in preschool age (grant CCM 2006).

Promotion of healthy lifestyles is also achieved through the study web-site (http://www.piccolipiu.it). To enhance communication with the participating families and within the community of participants the study has a Facebook page (Progetto Piccolipiù), where families can exchange information.

During the follow up, families are reminded of correct lifestyles, encouraged to visit the web site, to read the diaries and to complete the monthly forms on a monthly basis via SMS. Finally, a newsletter is periodically sent to the families and uploaded on the web site. Newsletters report updates on the project and convey health promotion messages.

### Ethical and legal procedures

The protocol of the study has been approved by the Ethics committees of the Local Health Unit Roma E (management centre), of the Istituto Superiore di Sanità (National Institute of Public Health) and of each local centre.

A consent form for participation is signed by the mother and also by the father, when both legally responsible for the newborn. It includes the consent to data collection and to follow up contact for interviews or medical examinations; it gives consent for the collection of the biological samples and their storage in the Biobank until the child is eighteen years old; it also gives consent for the use, in the future, of personal health data recorded in other health care registries as well as biochemical and genetic investigations. One copy of the consent form is given to families with details of the person to contact in case of withdrawal from the study or the Biobank, withdrawal being guaranteed at any time. If the consent is withdrawn by parents, both the data collected and the biological samples are destroyed in a timely manner.

Standard procedures for the protection of confidential individual information are applied according to the Italian law. Biological materials are collected, stored and used in accordance with national and international ethical recommendations and guidelines as well as national legal regulations.

### Monitoring and analysis plan

We plan to investigate several exposures and outcomes in order to provide an answer to a number of research questions, including:

– The relationship between maternal risk factors (such as overweight and obesity, weight gain during pregnancy, maternal complications in pregnancy and in particular maternal diabetes) and child obesity and early markers of cardiovascular disease;

– The association between social, psychological and economic determinants and child neuro-cognitive development and health, with particular focus on the detrimental effects of the economic crisis;

– The relationship between growth during early infancy (birth weight, weight gain and catch up growth) and occurrence of cardiovascular and metabolic outcomes (obesity, hypertension) or respiratory diseases (wheezing and asthma);

– The relationship between maternal complications in pregnancy, caesarean section and the presence of wheezing and asthma in children;

– The relationship between maternal vitamin D levels in pregnancy and respiratory infections, wheezing and asthma in childhood;

– The role of gene-environment interactions on specific health outcomes (e.g. respiratory diseases and child obesity);

– The identification of DNA methylation signatures in cord blood DNA associated with prenatal factors such as maternal infections, asthma and wheezing, pre and post pregnancy folate intake, and vitamin D level in pregnancy.

Piccolipiù proposes to be a resource for the research community. Data will be accessible to public researchers outside the Piccolipiù research group upon request for collaborative research initiatives, after approval by the Piccolipiù steering committee and clearance from the ethical committee.

### Power calculation

The Piccolipiù cohort is sized to have enough power to study relatively common child exposures and outcomes. Table 1 reports the minimum detectable relative risks for different scenarios of exposure prevalence considering an alpha error of 5%, an outcome with a prevalence of 10%, and a power of 80%. We also assume a 90% complete follow-up (implying a sample size of 2700 instead of 3000 children). Calculations in Table 1 refer to a dichotomous exposure and a dichotomous outcome, but power would be greater if continuous exposures and outcomes were considered.

**Table 1:** Minimum detectable relative risks for different scenarios of exposure prevalence

**Exposure prevalence**	**Relative risk**	**Examples**
5%	2.00	Association between maternal pre-pregnancy obesity and childhood obesity
10%	1.69	Association between maternal hypertension in pregnancy and asthma in childhood
25%	1.46	Association between lower socio-economic status and impaired cognitive development
50%	1.40	Association between gender and emergency department admittance for common diseases

Legend: Relative risks estimated for a cohort of 2700 children with complete follow-up for different scenarios of exposure prevalence, considering an 80% power, an alpha error of 5% and a risk of the outcome of 10%.

## Discussion

The Piccolipiù project is based on the joint effort and experience of public health researchers, epidemiologists and practicing clinicians involved in previous birth cohort research at national level in Italy.

The Piccolipiù birth cohort is a relatively large cohort combining detailed information from questionnaires and repeated follow-up contacts, with a biobank that stores samples from participating mother-newborn pairs. Collected data and biological samples will be made accessible to researchers outside the Piccolipiù group upon request for collaborative initiatives. Collaboration with other Italian and European cohorts is also foreseen. It is planned that the Piccolipiù cohort data will be linked and integrated with other data sources, such as routinely collected health data or data from other projects, in order to evaluate data quality and to test specific hypotheses. Questionnaires have been developed taking into account the comparability with other cohort studies, and using, as much as possible, validated scales or instruments.

Since loss to follow-up is always a cause for concern in cohort studies and should be minimized, efforts have been made to establish a close and trust-based relationship with the participating families. For the Piccolipiù project, ad-hoc developed diaries, SMS, study website, Facebook page and newsletters are being used to keep in touch with study participants. These tools are used to remind the participants the benefits of the study for the community, and to support participating families in child care. Through these instruments health promotion actions have been implemented within the cohort, as well as made available to the general population by the publication of recommendations on the website http://www.piccolipiu.it. An element of novelty is the use of web based resources to fill in the questionnaires, to stay in contact with the families and to convey health messages via the social networks (Facebook).

The Piccolipiù birth cohort will contribute to the understanding of early-life risk factors for child and adult health. Using a longitudinal approach, the study will be able to assess the harmful effects of worsening socioeconomic conditions on families, taking into account a number of cofactors, such as maternal emotional status, and monitoring changes in economic crisis-related data over time.

## Competing interests

The authors declare that they have no competing interests. The study received a grant by the Italian National Centre for Disease Prevention and Control (CCM grant 2010).

## Authors’ contributions

All the authors devoloped the study protocol, contributed to the drafting of the paper and approved it. All authors’ read and approved the final manuscript.

## Pre-publication history

The pre-publication history for this paper can be accessed here:

http://www.biomedcentral.com/1471-2431/14/36/prepub

## Supplementary Material

Additional file 1 Questionnaire main measures (from pregnancy to the age of 24 months). Click here for file
