# Supplementary material for: Piccolipiù, a multicenter birth cohort in Italy: protocol of the study
Source: BMC Pediatr. 2014 Feb 7;14:36. doi: 10.1186/1471-2431-14-36 (PMC3926689; doi:10.1186/1471-2431-14-36)
Supplement: Additional file 1 — Questionnaire main measures (from pregnancy to the age of 24 months). [file 1471-2431-14-36-S1.doc]

**Questionnaire main measures (from pregnancy to the age of 24 months)**

**Baseline questionnaire**

*Demographic data*: parental citizenship, years in Italy (if foreign), place of birth of grandparents.

*Social background*: parental occupations, parental educational level, employment.

*Household composition and Housing, indoor and outdoor pollution*: crowding (person/room), ownership, dimension, number of rooms, floor, presence of mould or moisture, availability of gas water heater, air conditioning, types of heating and cooking at home, air circulation, use of chemicals in the home, proximity to heavy traffic, pets.

*Leisure and life style*: maternal leisure and sport activity one year before pregnancy, maternal pre-pregnancy smoking and alcohol use, special nutritional needs, use of salt.

*Health*: paternal and maternal (pre-pregnancy) weight and height, maternal type of delivery, breastfeeding, menarche age and menstrual cycle regularity, parental weight at birth, parental morbidities, in particular diabetes and allergies; parental sleep pattern.

*Reproductive history*: previous reproductive outcomes, infertility, previous children breastfeeding, weight and gender, gestational age.

*Pregnancy*: emotional aspects (on pregnancy and in the last month using the GHQ-12, gestational week at the first visit, number of ultrasound check, antenatal diagnosis tests, other aspects (contact with pets, type of work, use of chemicals, sport activity, hours at open air, snore, active and passive smoking, alcohol use, diet); morbidities and drug use; hospital admission; vitamin supplementation; pregnancy weight gain.

*Delivery*: early breastfeeding, newborn sleep position.

**First follow-up questionnaire (6 months of life)**

*For each month of life (from birth to 6 months)*: weight and height, breastfeeding, weaning, hours of sleep, pacifier use, passive smoking.

*From birth to six months*: foods items with the month of introduction; morbidities and drugs; day nursery.

*Last 24 hours*: food items and quantity.

**Second follow-up questionnaire (12 months of life)**

*Child growth*: neurodevelopment assessment, weight and height.

*Child health*: diseases, hospital admittance, emergency room visits, drugs administered, unintentional injuries.

*Child care*: day nursery, mother and father activities with child, number of hours at open air per month.

*Family habits*: television viewing, maternal and paternal smoking habit, child passive smoking exposure.

*Child nutrition*: breast feeding, semi-quantitative food frequency.

*Child sleep*: questions from “brief screening questionnaire for infant sleep”.

*Socioeconomic position*: maternal and paternal occupational changes from birth to the first birthday, family income.

*Household composition and housing*: changes over time.

*Maternal mood*: mother-to-infant attachment scale, general health questionnaire (12 items), positivity scale.

**Third follow-up questionnaire (24 months of life)**

*Child growth*: neurodevelopment assessment, weight and height.

*Child health*: diseases, hospital admittance, emergency room visits, drugs administered, unintentional injuries.

*Child care*: day nursery, mother and father activities with child.

*Child activities*.

*Family life style*: maternal and paternal smoking habit, child passive smoking exposure.

*Child nutrition*: breast feeding, dietary patterns and feeding style.

*Child sleep*: questions from “brief screening questionnaire for infant sleep”.

*Socioeconomic position*: maternal and paternal occupational changes from the last follow-up.

*Household composition and housing*: changes over time.
